# Comparison of cognitive and brain grey matter volume profiles between multiple sclerosis and neuromyelitis optica spectrum disorder

**DOI:** 10.1371/journal.pone.0184012

**Published:** 2017-08-28

**Authors:** Hiroki Masuda, Shigeki Hirano, Nobuyoshi Takahashi, Etsuko Hatsugano, Akiyuki Uzawa, Tomohiko Uchida, Ryohei Ohtani, Satoshi Kuwabara, Masahiro Mori

**Affiliations:** 1 Department of Neurology, Graduate School of Medicine, Chiba University, Chiba, Japan; 2 Department of Rehabilitation Sciences, Chiba Prefectural University of Health Sciences, Chiba, Japan; 3 School of Medicine, Chiba University, Chiba, Japan; Nagoya Daigaku, JAPAN

## Abstract

**Objective:**

Brain regions responsible for cognitive dysfunction in MS and neuromyelitis optica spectrum disorder (NMOSD) are not known. Our aim of this study was to investigate whether cognitive function and brain volume differed between MS and NMOSD in Japanese patients.

**Methods:**

Brain MRI and neuropsychological tests including the Wechsler Adult Intelligence Scale-III (WAIS-III), Wechsler Memory Scale-Revised (WMS-R), Trail Making Test (TMT) and Clinical Assessment for Attention (CAT) were performed. Parametric grey matter (GM) and white matter (WM) volumes determined from lesion-filled T1-weighted images using whole-brain voxel-based morphometry (VBM) were compared by two-tailed *t* test.

**Results:**

Twenty relapsing-remitting MS and sixteen NMOSD patients were included. MS patients were younger than NMOSD patients. Processing speed intelligence quotient (IQ), general memory, verbal memory and delayed recall were significantly worse in MS patients than in NMOSD patients. Furthermore, left superior temporal gyrus (STG) GM volume was smaller in MS patients than in NMOSD patients (*P* < 0.05, family-wise error [FWE] corrected, Z_max_ = 4.97, 62 voxel). The left STG GM volume tended to be positively correlated with delayed recall in MS patients.

**Conclusions:**

Despite being younger, MS patients demonstrated worse performance in certain cognitive variables than NMOSD patients, which might be associated with left STG GM volume loss.

## Introduction

Cognitive dysfunction is reported in 43%–65% of patients with MS [1] and is strongly correlated with white matter (WM) lesion volume and brain atrophy. Altered memory and information processing speed were recently identified as the most common dysfunctions in MS patients. On the other hand, it was reported that some MS patients suffered from significant dysfunction in visuospatial abilities and executive functioning, but the frequency was not so high. [1] In addition, neuropsychological test scores were shown to correlate only weakly with disease duration and neurological disability. [1]

Recently, changes in cognitive function of patients with neuromyelitis optica spectrum disorder (NMOSD) were reported by several groups. The first study by Blanc and colleagues used the Brief Repeatable Battery of Neuropsychological Tests (BRB-N) as well as three additional tests to compare 30 MS and 30 NMO patients with 30 healthy controls (HCs). [2] The study reported that the cognitive impairment in MS and NMO patients was significantly worse than that in HCs; however, they did not find a difference in cognitive function between MS and NMO patients. Another study comparing cognitive function and depression in MS and NMOSD patients and HCs (n = 42 per group) found that cognitive impairment was experienced by 67% of the NMOSD patients, whereas the prevalence of cognitive impairment was similar between patients with MS and those with NMOSD. [3] Saji et al. investigating BRB-N in a cohort of patients with MS (n = 17) or NMOSD (n = 14) and HCs (n = 37) reported that cognitive dysfunction was observed in 57% and 47% of NMOSD and MS patients, respectively, with similar cognitive profiles between the two disease groups. [4]

Brain grey matter (GM) and WM volumes in patients with MS and NMOSD were also assessed by voxel-based morphometry (VBM). [5–7] Prinster and colleagues reported significant GM volume loss in relapsing-remitting MS patients compared to age-matched HCs. [5] One VBM study revealed that deep GM volume was significantly lower in patients with MS than in those with NMO. [6] Yet another study reported that the GM and total brain volume were lower in MS patients than in NMO patients, whereas the WM volume did not differ between MS and NMO. [7]

Collectively, the results from several studies indicated that MS and NMOSD patients did not show a difference in cognitive, whereas some VBM studies reported that MS patients exhibited GM volume loss compared to patients with NMOSD. These divergent results highlight the need for further assessment to draw clear conclusions on the difference in cognitive function between MS and NMOSD given that GM volume loss could lead to cognitive dysfunction. The first aim of this study was to investigate potential differences in cognitive function and regional GM volume between MS and NMOSD patients. Second, we aimed to identify the specific brain region responsible for cognitive impairment in MS and NMOSD patients by analysis of correlation between cognitive function and regional GM volume.

## Materials and methods

### Patients

In this study, 20 relapsing-remitting MS patients and 16 NMOSD patients were included. All MS patients fulfilled the 2010 Revised McDonald criteria, [8] and all NMOSD patients fulfilled the 2015 international consensus diagnostic criteria. [9] Subjects who relapsed within three months of brain MRI or neuropsychological tests and those who were unable to perform neuropsychological tests because of severe bilateral visual loss or disturbance in dominant upper extremities were excluded. In this study, ethical approval was obtained from the ethics committee of Chiba University School of Medicine. All patients provided informed consent.

### Clinical characteristics

We reviewed age at disease onset and sex as well as the following parameters at the time of brain MRI: age and disease duration, number of attacks, Kurtzke Expanded Disability Status Scale (EDSS), presence of brain MRI abnormalities suggestive of MS or NMOSD, functional visual acuity score, years of education, anti-aquaporin-4 antibody positivity [10] and treatment.

### Evaluation of cognitive function

Neuropsychological tests were performed within six months of brain MRI scans and included the Wechsler Adult Intelligence Scale-III (WAIS-III), Wechsler Memory Scale Revised version (WMS-R), Trail Making Test (TMT) and Clinical Assessment for Attention (CAT).

### Brain MRI

Conventional brain MRI, T1-weighted three-dimensional images and fluid-attenuated inversion recovery (FLAIR) were obtained from all participants. Three MR systems used in this study were 1.5-Tesla Signa HDxT (GE Healthcare, Milwaukee, Wisconsin, USA), 3.0-Tesla Discovery MR 750 (GE Healthcare) and 1.5-Tesla Achieva (Philips, Amsterdam, NL) scanners. Signa HDxT, Discovery MR 750 and Achieva scanners were used in 16, 3 and 1 patient with MS and in 10, 4 and 2 patients with NMOSD, respectively. For Signa HDxT, we obtained T1-weighted three-dimensional images in sagittal plane (field of view [FOV], 240 mm × 240 mm; number of sections, 248; section thickness, 1.4 mm; repetition time [TR], 7 ms; echo time [TE], 2.9–3.0 ms; inversion time [TI], 0 to 420 ms; number of signals acquired, 0.9921 to 1; echo train length, 1; flip angle [FA], 15°) and FLAIR (FOV, 220 mm × 220 mm; number of sections, 32; section thickness, 4 mm; TR, 10000–10002 ms, TE, 120.2–128.6 ms, TI, 2400 ms; number of signals acquired, 1; echo train length, 1; FA, 90°). Details of the other two MRI systems are shown in S1 Table.

### Brain grey matter and white matter volume measurements

Regional GM and WM volume changes were determined by the Lesion Segmental Tool (LST) developed by Schmidt et al. (http://www.applied-statistics.de/lst.html) and voxel-based morphometry eight (VBM8; http://dbm.neuro.uni-jena.de/vbm/). [11] [12] Briefly, LST automatically identified plaques on FLAIR images and filled T1 black holes on T1-weighted three-dimensional images. Subsequently, segmentation and spatial normalization by diffeomorphic anatomical registration through exponentiated lie algebra (DARTEL) were achieved by VBM8. Initial threshold (κ) values for lesion filling were optimized as below. First, lesion-filled-images were prepared from κ values of 0.15 to κ values of 0.50 with interval κ values of 0.05. Three MS specialists separately voted for the κ value of 0.20 which used for lesion filling, which was followed by segmentation using VBM8. We compared differences in GM and WM volumes between MS and NMOSD patients using statistical parametric mapping eight (SPM8) and two-tailed *t* test with age as a covariate, implemented on Matlab version R2012b on Windows 7. Intracranial volume (ICV), calculated by the sum of whole brain GM, WM and cerebrospinal fluid volumes, was treated as a covariate to normalize for head size. Lesion volume filled by LST was expressed as total lesion volume (TLV).

### Calculation of volume of interest

Volume-of-interest (VOI) was automatically calculated as sphere of radius six mm from a peak coordinate significantly different in MS and NMOSD derived from two-tailed t-test with SPM8 and subsequently normalized by ICV.

#### Statistical analysis

Continuous data were compared using the Mann-Whitney *U* test. Categorical outcomes were evaluated using the chi-square test or Fisher’s exact test. Spearman’s rank test was performed to analyse correlations. *P* values of <0.05 were considered statistically significant. For VBM analysis, height threshold of *P* < 0.05 corrected for family-wise error (FWE) and extent threshold at least 50 voxels were considered significant. Coordinates were reported in the standard anatomical space developed at the Montreal Neurological Institute (MNI). [13] Statistical tests were conducted using SPSS^®^ version 22.0 (IBM Corporation, Armonk, NY, USA).

## Results

### Demographics, clinical characteristics and neuropsychological performance of MS and NMOSD patients

Demographics, clinical characteristics and the results of neuropsychological tests of the study cohort are shown in Tables 1 and 2. Median age of MS patients at the time of brain MRI scans was lower than that of NMOSD patients (38.5 versus 55.0 years, *P* = 0.012). Although the percentage of brain MRI scans suggestive of MS was significantly higher in MS patients than in NMOSD patients (95% versus 40%, *P* = 0.005), the percentages of brain MRI scans suggestive of NMOSD were not different between the two groups. Meanwhile, median functional visual acuity score of NMOSD patients at the time of brain MRI was higher than that of MS patients (0.0 versus 2.0, *P* = 0.016). Other clinical characteristics including age at disease onset; sex; disease duration, number of attacks and EDSS at the time of brain MRI; and years of education were not different between the MS and NMOSD patients. None of the MS patients were positive for anti-aquaporin-4 antibody, whereas anti-aquaporin-4 antibody positivity was observed in all but one NMOSD patients. Treatment with interferon-β was significantly more frequent among MS patients than NMOSD patients at the time of brain MRI (P = 0.005). Conversely, prednisolone therapy was more frequently used in patients with NMOSD than in those with MS at the time of brain MRI (*P* < 0.001). Evaluation of patients with WAIS-III revealed that median processing speed intelligence quotient (IQ) was lower in MS patients than in NMOSD patients (79.5 versus 92.0 years, *P* = 0.036). There were no differences in CAT and TMT scores between the two patient groups. Assessment by WMS-R determined that general memory, median verbal memory and delayed recall scores were lower in MS patients than in NMOSD patients (95.0 versus 115.5, *P* = 0.024; 91.0 versus 117.0, *P* = 0.012; 87.0 versus 111.0, *P* = 0.013; respectively).

**Table 1:** Demographic and clinical characteristics, and laboratory findings in MS and NMOSD patients.

	MS (n = 20)	NMOSD (n = 16)	*P*
**Demographic and clinical features**			
Female	14 (70%)	15 (94%)	0.10
Age at disease onset (years)	30.0 [7.3]	37.0 [24.8]	0.19
Age at brain MRI scan	38.5 [15.3]	55.0 [16.0]	0.012*
Disease duration to brain MRI scan (years)	6.5 [9.3]	8.0 [15.3]	0.18
Number of attacks to brain MRI scan	4.0 [5.3]	4.0 [7.0]	0.88
EDSS at brain MRI scan	4.0 [2.8]	3.0 [4.0]	0.82
FS scores of visual acuity at brain MRI	0.0 [0.0]	2.0 [5.0]	0.016*
Brain MRI findings suggestive of MS	19 (95%)	8 (40%)	0.005*
Brain MRI findings suggestive of NMOSD	2 (10%)	2 (10%)	1.0
Years of education	14.0 [4.0]	12.0 [2.0]	0.22
**Laboratory findings**			
Patients with positive antibodies against aquaporin-4	0 (0%)	15 (94%)	<0.001*
**Treatments at the brain MRI**			
Any immunomodulating treatment	14 (70%)	13 (81.3%)	0.70
Interferon-β	8 (40%)	0 (0%)	0.005*
Fingolimod	5 (25%)	0 (0%)	0.053
Continuous oral prednisolone	0 (0%)	13 (81.3%)	<0.001*
Azathioprine	0 (0%)	2 (12.5%)	0.19

Data are presented as median [interquartile range] or number (%).

*P < 0.05.

EDSS: Kurtzke’s Expanded Disability Status Scale; FS: functional systems; NMOSD: neuromyelitis optica spectrum disorder.

**Table 2:** Neuropsychological test results in MS and NMOSD patients.

	MS (n = 20)	NMOSD (n = 16)	*P*
**Neuropsychological tests**			
**WAIS-III**			
Verbal IQ	95.5 [21.8]	102.0 [24.8]	0.25
Performance IQ	84.0 [24.3]	94.5 [18.5]	0.078
Full scale IQ	88.0 [26.8]	98.0 [22.0]	0.11
Verbal comprehension IQ	95.0 [11.5]	101.0 [22.0]	0.19
Perceptual organization IQ	89.0 [24.3]	92.0 [25.3]	0.38
Working memory IQ	88.0 [34.3]	94.0 [18.3]	0.48
Processing speed IQ	79.5 [29.8]	92.0 [25.8]	0.036*
**CAT**			
Visual cancellation 3 (% correct answer)	99.0 [1.0]	99.0 [3.0]	0.49
Visual Cancellation "か"** (% correct answer)	98.0 [7.0]	96.5 [6.3]	0.39
Visual Cancellation 3 (Completion Time)	108.5 [39.5]	100.0 [42.8]	0.99
Visual Cancellation "か"** (Completion Time)	125.0 [56.5]	110.0 [61.0]	0.73
Auditory Detection (% correct answer)	100.0 [4.0]	98.0 [4.0]	0.38
Auditory Detection (Accuracy)	100.0 [4.0]	100.0 [0.0]	0.22
PASAT 2 sec (% correct answer)	57.0 [46.0]	57.5 [50.0]	0.88
PASAT 1 sec (% correct answer)	33.5 [26.8]	34.0 [23.5]	0.68
SDMT (Achievement rate)	42.5 [34.8]	43.5 [22.0]	0.60
**WMS-R**			
General memory	95.0 [26.8]	115.5 [27.3]	0.024*
Verbal memory	91.0 [27.0]	117.0 [27.3]	0.012*
Visual memory	104.0 [33.5]	108.5 [18.8]	0.23
Attention/Concentration	96.5 [22.8]	98.0 [28.0]	0.91
Delayed recall	87.0 [37.5]	111.0 [25.5]	0.013*
**TMT**			
Part A (second)	44.0 [37.0]	38.5 [29.5]	0.13
Part A (The number of error)	0 [0]	0 [0]	1.0
Part B (second)	102.0 [93.0]	102.5 [84.3]	0.60
Part B (The number of error)	0 [0]	0 [1.0]	0.059

Data are presented as median [interquartile range] or number (%).

*P < 0.05.

CAT, Clinical Assessment for Attention; IQ, intelligence quotient; NMOSD, neuromyelitis optica spectrum disorder; PASAT, Paced auditory serial addition test; WAIS, Wechsler Adult Intelligence Scale; WMS-R, Wechsler Memory Scale-Revised; SDMT, Symbol digit modalities test; TMT, Trail Making Test.

### Significant differences in correlations among neuropsychological test scores between MS and NMOSD patients

Among MS patients, all components of the neuropsychological test scores that were significantly different between the two groups (i.e. scores for processing speed IQ, general memory, verbal memory and delayed recall) showed positive correlations (S2 Table). Specifically, any combination of two of the four tests were significantly correlated. For example, verbal memory was positively correlated with delayed recall (rho, 0.91; *P* < 0.001), general memory (rho, 0.96; *P* < 0.001) and processing speed IQ (rho, 0.73; *P* < 0.001). Delayed recall also showed positive correlations with general memory (rho, 0.96, *P* < 0.001) and processing speed IQ (rho, 0.74; *P* < 0.001), whereas general memory was also positively correlated with processing speed IQ (rho, 0.66; *P* = 0.001). Conversely, positive correlations were also found among all factors other than processing speed IQ in NMOSD patients (S2 Table). In fact, verbal memory showed positive correlations with delayed recall (rho, 0.99; *P* < 0.001) and general memory (rho, 0.98; *P* < 0.001) but not with processing speed IQ (rho, 0.33; *P* = 0.21). Delayed recall was also positively correlated with general memory (rho, 0.98; *P* < 0.001) but not with processing speed IQ (rho, 0.39; *P* = 0.14). Finally, there was no correlation between general memory and processing speed IQ (rho, 0.43; *P* = 0.097) in NMOSD patients.

### Comparison of lesion volumes between MS and NMOSD patients

TLV, ICV and TLV/ICV in MS and NMOSD patients are shown in Table 3. Although TLV/ICV in MS patients tended to be higher than those of NMOSD patients, there was no statistically significant difference in median TLV/ICV values between the two groups (0.015 versus 0.0048, *P* = 0.077).

**Table 3:** TLV, ICV, and TLV/ICV in MS and NMOSD patients.

	MS(n = 20)	NMOSD(n = 16)	*P*
TLV	18.1 [37.3]	5.94 [24.9]	0.077
ICV	1260 [167]	1280 [109]	0.67
TLV/ICV	0.147 [0.0282]	0.0484 [0.178]	0.077

Data are presented as median [interquartile range].

ICV: intracranial volume; TLV: total lesion volume; NMOSD: neuromyelitis optica spectrum disorder.

### Comparison of brain volumes of MS and NMOSD patients

**Fig 1:**
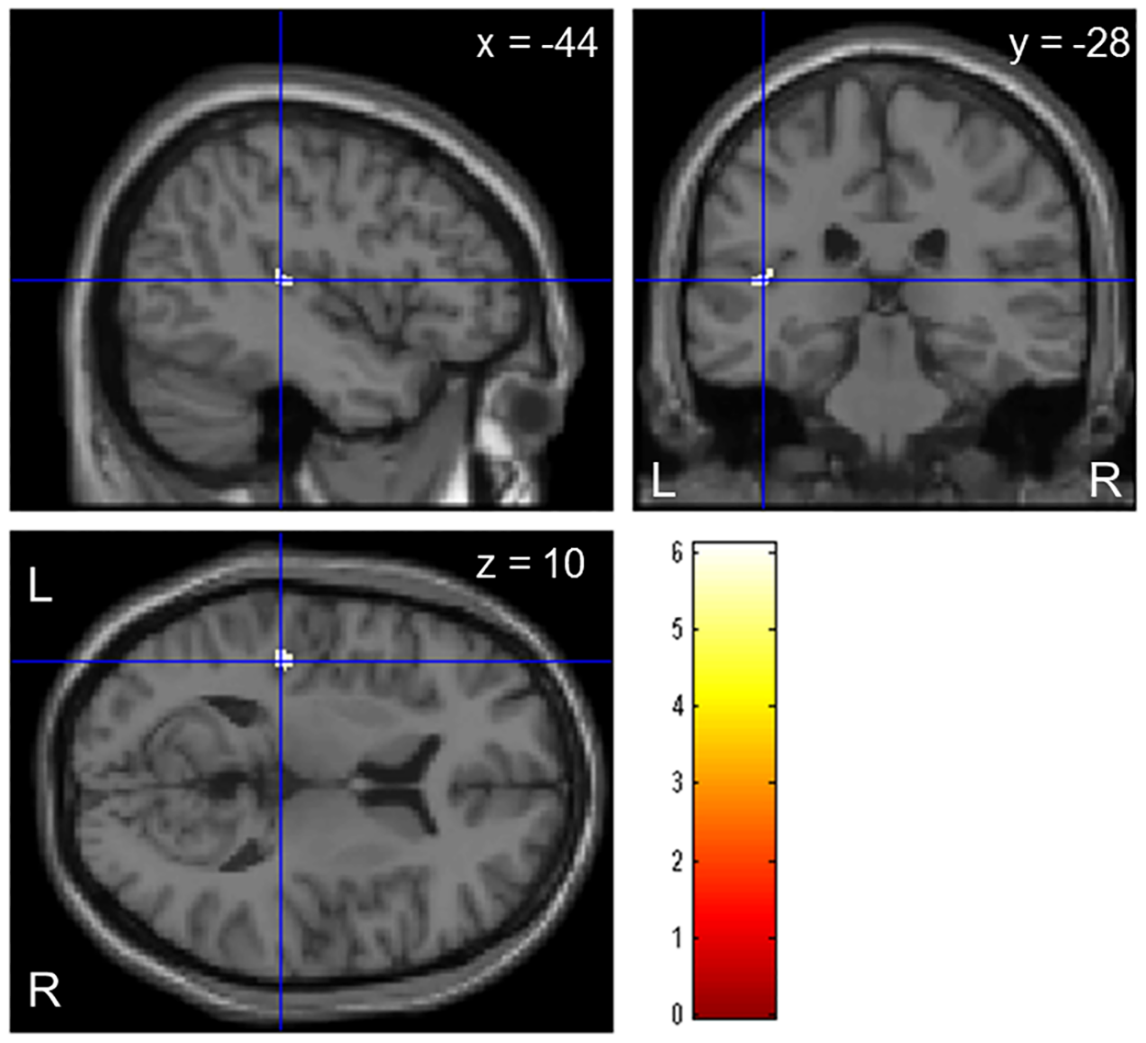
Voxel-based morphometry analysis in patients with multiple sclerosis and neuromyelitis optica spectrum disorder. Statistical parametric map of differences in grey matter volume between 20 multiple sclerosis and 16 neuromyelitis optica spectrum disorder patients (*P* < 0.05, family-wise error corrected). Regions with smaller grey matter were located in left superior temporal gyrus in multiple sclerosis patients. Data are superimposed on a standard single case T1-weighted image with statistical parametric mapping 8. Cluster is shown with red-yellow colour coding.

Analysis of brain MRI scans with VBM showed that GM volume of the left superior temporal gyrus (STG) was smaller in MS patients than in NMOSD patients (FWE corrected *P* < 0.05, Z_max_ = 4.97, 62 voxel, peak MNI coordinates: −44, −28, 10; Fig 1). Conversely, there was no difference in WM volumes between the two groups. Three MRI systems were used to obtain brain scans in this study; therefore, we also performed the same analysis in patients who underwent brain MRI on Signa HDxT, which was used in the majority of cases and found that GM volumes of the left STG and posterior cingulate gyrus were smaller in MS patients than in NMOSD patients (FWE corrected *P* < 0.05, Z_max_ = 5.49 and 5.87, 503 and 176 voxels, peak MNI coordinates: −44, −22, 7 located in Brodmann's area 13 and −9, −58, 3 in Brodmann's area 30, respectively). Furthermore, we obtained the same result by using age and MRI scanner difference as covariates.

### Correlations between the left STG volume and neuropsychological test scores

**Fig 2:**
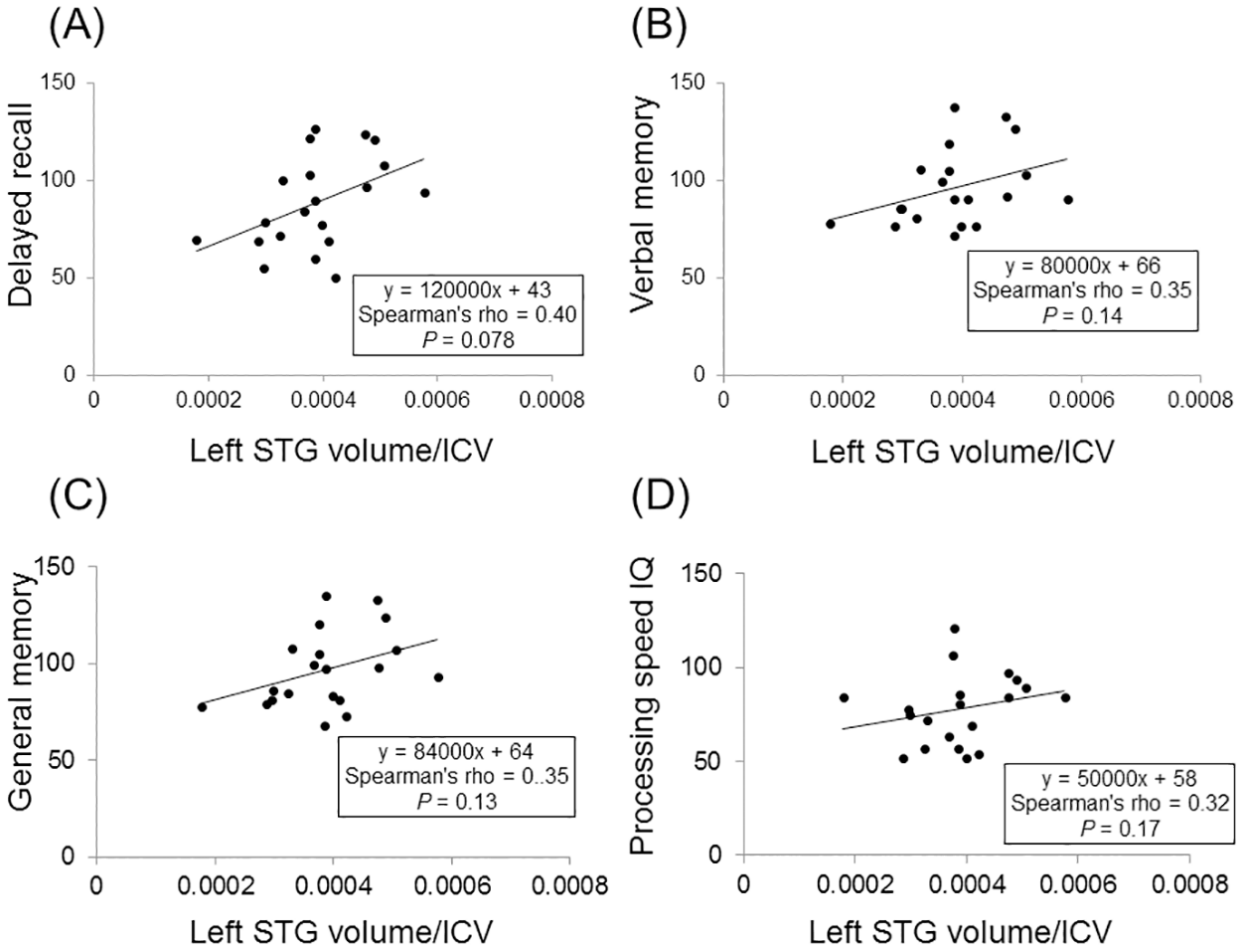
Correlations between left superior temporal gyrus (STG) volume and neuropsychological test scores in multiple sclerosis patients. Correlations were analysed by Spearman’s rank correlation test. Intracranial volume (ICV) was calculated by sum of whole brain grey matter, white matter and cerebrospinal fluid volumes. Left STG volume was divided by ICV to adjust for the head size of each patient. Results of correlation analyses are shown for (A) left STG volume and verbal memory, (B) left STG volume and delayed recall, (C) left STG volume and general memory and (D) left STG volume and processing speed intelligence quotient (IQ). No statistically significant correlations were found in any of the combinations. However, a trend towards a positive correlation was found between left STG volume and delayed recall.

First, we investigated correlations between the left STG volume and neuropsychological test scores differed in the two groups. Specifically, analysis in MS patients determined that the left STG volume tended to be correlated with delayed recall (rho = 0.40, *P* = 0.078) (Fig 2A), whereas verbal memory, general memory and processing speed IQ were not correlated with the left STG volume (rho < 0.35, *P* > 0.13) (Fig 2B–2D). In NMOSD patients, no correlations were found between the four tests and the left STG volume (*P* > 0.68). Next, correlations between the left STG volume and neuropsychological test scores not differed in the two groups were investigated. In MS patients, the STG volume was positively correlated with accuracy of auditory detection (rho = 0.51, *P* = 0.027) and symbol digit modalities test (SDMT; rho = 0.57, *P* = 0.015). The time to finish TMT part B was negatively correlated with the left STG volume in MS patients (rho = −0.61, *P* = 0.006). On the other hand, both the percentage of correct answers in visual cancellation 3 and SDMT were positively correlated with the STG volume in NMOSD patients (rho = 0.51 and 0.50, *P* = 0.043 and 0.047, respectively).

### Correlation between TLV/ICV and neuropsychological test scores in MS and NMOSD patients

TLV/ICV was negatively correlated with several neuropsychological test scores in MS patients including verbal IQ (rho = −0.52, *P* = 0.019), full scale IQ (rho = −0.46, *P* = 0.043), verbal comprehension IQ (rho = −0.52, *P* = 0.018), processing speed IQ (rho = −0.70, *P* = 0.001), accuracy of auditory detection (rho = −0.55, *P* = 0.015), SDMT (rho = −0.66, *P* = 0.003), general memory (rho = −0.46, *P* = 0.042), verbal memory (rho = −0.45, *P* = 0.046), visual memory (rho = −0.46, *P* = 0.042) and delayed recall (rho = −0.48, *P* = 0.031). In contrast, time to finish visual cancellation 3, time to finish drawing ‘か’ which is the letter ‘ka’ in Japanese ‘hiragana’ characters and times to finish TMT parts A and B were positively correlated with TLV/ICV in MS patients (rho = 0.53, 0.59, 0.54 and 0.64, *P* = 0.024, 0.010, 0.018 and 0.003, respectively). Conversely, in NMOSD patients, TLV/ICV was negatively correlated with accuracy of visual cancellation 3 (rho = −0.55, *P* = 0.027) and SDMT (rho = −0.63, *P* = 0.009). Positive correlation was found only between TLV/ICV and time to finish TMT part A (rho = 0.59, *P* = 0.015).

## Discussion

The current study revealed the following: 1) MS patients exhibited worse processing speed IQ, verbal memory, general memory and delayed recall than NMOSD patients despite their relatively younger age; 2) GM volume of the left STG was smaller in MS patients than in NMOSD patients. 3) The left STG volume tended to be correlated with delayed recall.

The current study demonstrated that processing speed IQ, evaluated by visual attention in WAIS-III, was lower in MS patients than in NMOSD patients. However, other tests used to determine visual attention such as CAT and TMT were not different between the two groups. Therefore, based on the incongruent results obtained by processing speed IQ, CAT and TMT, our findings regarding visual attention should be interpreted with caution.

Our study also showed MS patients demonstrated worse general memory in WMS-R than NMOSD patients. As general memory in WMS-R consists of verbal memory and visual memory, decreased general memory may stem from decreased verbal memory in MS patients. Another study reported that MS patients showed worse verbal and visual memory, and verbal learning compared with NMOSD patients. [14] Both our study and the study performed by Kim et al., showed decreased verbal memory in MS patients compared with NMOSD patients. However, visual memory was not different between MS and NMOSD patients in our study. Therefore, decreased verbal memory may be a feature distinguishing cognitive impairment in MS patients from those in NMOSD patients.

Previous VBM studies reported that the left STG volume was smaller in MS patients than in HCs. [5] [15] Achiron et al. reported that the left STG thickness correlated with attention and information processing, in line with the current observation. [15]

Left STG is generally associated with auditory and speech comprehension, [15] whereas delayed recall consists of visual and verbal elements. Therefore, decreased auditory and speech comprehension could cause deficits in both verbal memory and delayed recall. Although auditory and speech comprehension were not fully assessed in the current study, verbal comprehension in WAIS-III was not different between MS and NMOSD patients. Therefore, further investigation is required to confirm our hypothesis that deficits observed in verbal memory and delayed recall observed in MS patients might stem from worse auditory and speech comprehension experienced by MS patients compared with NMOSD patients.

This study has several limitations. First, brain MRI scans were performed by three instruments at random. However, similar coordinates were obtained even when the analysis was limited to the group of patients who were analysed with the same scanner, Signa HDxT. In addition, we obtained the same result by adding not only age but also MRI scanner difference as covariates. Therefore, we considered MRI scanner difference had little effect on the results. Second, HCs were not included in this study. Finally, not only the smaller left STG volume but also the tendency for larger TLV/ICV in MS patients may affect neuropsychological test results.

## Conclusions

In conclusion, MS patients exhibited worse verbal memory and delayed recall than NMOSD patients regardless of their relatively younger age. GM volume loss in the left STG in MS patients might be associated with cognitive impairment.

## Supporting information

S1 Table Details of other two MRI systems. (DOCX)

S2 Table Correlations among cognitive function significantly different in patients with MS and NMOSD. (DOCX)
