# Supplementary material for: Comparison of cognitive and brain grey matter volume profiles between multiple sclerosis and neuromyelitis optica spectrum disorder
Source: PLoS One. 2017 Aug 28;12(8):e0184012. doi: 10.1371/journal.pone.0184012 (PMC5573289; doi:10.1371/journal.pone.0184012)
Supplement: S1 Table — (DOCX) [file pone.0184012.s001.docx]

Supplementary Table 1. Details of other two MRI systems.

|  | Discovery MR 750 | Achieva |  |
| --- | --- | --- | --- |
| Tesla  3DT1  FOV    Number of sections  Section thickness  TR  TE  TI  Number of signals acquired  Echo train length  FA  FLAIR  FOV  Number of sections  Section thickness  TR  TE  TI  Number of signals acquired  Echo train length  FA | 3.0  220mm × 220mm  to 256mm × 256mm  178 to 248  1.0mm to 1.4mm  7ms to 8ms  3.0ms to 3.3ms  420ms  0.5 to 1  1  15°  220mm × 220mm  32  4mm  10000ms  131.2-131.8ms  2336.49ms to 2500ms  1  1  90° to 111° | 1.5  230mm × 230mm  127 to 128  1.5mm  22ms  4.6ms  NA  1  1  25°  220mm × 220mm  32  4mm  11000ms  120ms  2800ms  2  38  90° |  |

**P* < 0.05. 3DT1: T1-weighted three-dimensional images; FA: flip angle; FLAIR: fluid attenuated inversion recovery; FOV: field of view; NA: not acquired; TR: repetition time; TE: echo time; TI: inversion time.
