# Supplementary material for: Comparison of cognitive and brain grey matter volume profiles between multiple sclerosis and neuromyelitis optica spectrum disorder
Source: PLoS One. 2017 Aug 28;12(8):e0184012. doi: 10.1371/journal.pone.0184012 (PMC5573289; doi:10.1371/journal.pone.0184012)
Supplement: S2 Table — (DOCX) [file pone.0184012.s002.docx]

Supplementary Table 2. Correlations among cognitive function significantly different in patients with MS and NMOSD.

|  | Spearman’s rho  in MS | *P* | Spearman’s rho  in NMOSD | *P* |
| --- | --- | --- | --- | --- |
| Verbal memory vs. Delayed recall  Verbal memory vs. General memory  Verbal memory vs. Processing speed IQ  Delayed recall vs. General memory  Delayed recall vs. Processing speed IQ  General memory vs. Processing speed IQ | 0.91  0.96  0.73  0.96  0.74  0.66 | <0.001*  <0.001*  <0.001*  <0.001*  <0.001*  0.001* | 0.99  0.98  0.33  0.98  0.39  0.43 | <0.001*  <0.001*  0.21  <0.001*  0.14  0.097 |

**P* < 0.05. IQ: intelligence quotient; MS: multiple sclerosis; NMOSD: neuromyelitis optica spectrum disorder.
